# RNA Binding Protein YTHDF2 Inhibits Synovial Fibroblast Inflammation and Bone Injury in Rheumatoid Arthritis by Reducing the mRNA Stability of IL‐6R

**DOI:** 10.1002/kjm2.70010

**Published:** 2025-06-23

**Authors:** Yan Zhang, Xu‐Qing Cao, Xiao‐Li Ma, Yin‐Yan Guo, Tao Zhang, Li‐Li Wu, Ya‐Shan Yang, Chun‐Fang Hao, Wei‐Li Liu, Jiang‐Tao Guo

**Affiliations:** ^1^ Department of Rheumatology and Immunology People's Hospital of Ningxia Hui Autonomous Region (The Third Affiliated Hospital of Ningxia Medical University) Yinchuan City China; ^2^ Department of Neurology People's Hospital of Ningxia Hui Autonomous Region (The Third Affiliated Hospital of Ningxia Medical University) Yinchuan China; ^3^ Department of Neurology Baoan District Central Hospital Shenzhen City China; ^4^ Department of Rheumatology and Immunology The Second Affiliated Hospital of the Chinese University of Hong Kong, Shenzhen (Longgang District People's Hospital of Shenzhen) Shenzhen China; ^5^ Department of Rheumatology and Immunology Baoan District Central Hospital Shenzhen China

**Keywords:** fibroblast‐like synoviocytes, IL‐6R, inflammation, rheumatoid arthritis, YTHDF2

## Abstract

This study examined YTHDF2's role in modulating IL‐6R signaling to regulate synovial fibroblast inflammation and bone damage in rheumatoid arthritis (RA). Synovial tissues of RA patients were collected. Human fibroblast‐like synoviocyte (FLS), MH7A cell line, was induced with TNF‐α and transfected. Cell proliferation was assessed using MTT and EdU assays; apoptosis was measured with flow cytometry, and migration and invasion were evaluated through scratch and Transwell assays. Lentiviral vectors designed to overexpress YTHDF2 or IL‐6R were created to study their effects in mice with collagen‐induced arthritis (CIA). Pathological changes of ankle joints in mice were observed, and TNF‐α, IL‐1β, and IL‐6 contents were determined. MMP3 and MMP9 levels were detected by Western blot, while YTHDF2 and IL‐6R were detected by RT‐qPCR and Western blot. The binding relationship between YTHDF2 and IL‐6R was studied. YTHDF2 in synovial tissues of RA patients was down‐regulated. Elevating YTHDF2 inhibited TNF‐α‐induced MH7A cell proliferation, migration, invasion, and pro‐inflammatory factors; Knocking down YTHDF2 showed the opposite effect. Upregulating YTHDF2 improved synovial inflammation and bone damage in CIA mice. IL‐6R in synovial tissues of patients was significantly up‐regulated and negatively correlated with YTHDF2 expression. YTHDF2 reduced IL‐6R mRNA stability in a m6A‐dependent manner. Overexpressing IL‐6R impaired the anti‐proliferating and anti‐inflammatory effect of YTHDF2 on TNF‐α‐induced MH7A cells. In CIA mice, overexpression of IL‐6R reversed the benefits on synovial inflammation and bone injury mediated by up‐regulating YTHDF2. YTHDF2 inhibits inflammation and bone damage in RA synovial fibroblasts by reducing the mRNA stability of IL‐6R.

## 1 Introduction

Rheumatoid arthritis (RA) is a chronic autoimmune disease [1]. The primary pathological alterations include chronic synovitis that is persistent and recurrent, resulting in significant inflammatory cell infiltration, pannus formation, and the destruction of intra‐articular cartilage and bone, ultimately causing joint dysfunction [2, 3]. The synovial membrane primarily consists of two types of cells: fibroblast‐like synoviocytes (FLS) and a few macrophage‐like synoviocytes. The excessive proliferation of FLS leads to the proliferation of synovial tissue and mediates the inflammatory response. The immune system in RA‐diseased joints produces inflammatory cytokines, which promote FLS proliferation and activation, ultimately leading to joint destruction [4]. RA FLS at the pannus‐cartilage interface increase the production of matrix metalloproteinases (MMPs), thereby degrading the extracellular matrix and destroying the collagen‐rich structure in the joints [5, 6]. RA FLS also show tumor‐like phenotypes, including aggressive proliferation and invasion, which greatly promote pannus formation and joint destruction [7, 8]. Despite some progress in the study of RA synovial inflammatory mechanisms, the molecular mechanisms underlying RA FLS activation and its mediated overproduction of inflammatory factors have not been systematically elucidated.

The abnormal biological behavior of RA FLS is significantly influenced by genetic and epigenetic regulation [9, 10, 11]. N6‐methyladenosine (m6A) is a significant RNA modification in eukaryotic cells that affects RNA processing, translation, and degradation, playing a role in cellular differentiation and tissue development [12, 13]. This dynamic and reversible modification is mainly regulated by methyltransferase complexes, demethylases, and binding proteins [14]. YTH‐domain family proteins (YTHDF) are important reading proteins for m6A methylation and translation regulation [15]. The YTHDF2 protein binds to target mRNAs and affects the stability of those mRNAs, regulating gene expression as a result [16, 17]. The pathophysiology of RA depends heavily on m6A modification [18, 19]. YTHDF2 has also been found to be expressed abnormally in RA and is closely associated with RA inflammation. It regulates inflammatory factor levels and invasive symptoms in RA FLS [20, 21, 22]. Nevertheless, the precise mechanism for YTHDF2 in RA has not been fully understood.

In RA, proinflammatory cytokines (TNF‐α, IL‐1, and IL‐6, etc.) initiate and maintain joint inflammation and destruction [23]. The cytokine IL‐6 has pleiotropic effects on immune responses, hematopoiesis, acute phase responses, and inflammation. It exerts a variety of biological activities through a hexamer complex composed of IL‐6 itself, receptor IL‐6R, and glycoprotein 130 [24, 25]. As a result of infections or tissue damage, IL‐6 is produced rapidly, resulting in increased acute phase proteins and immune response activation. Excessive IL‐6 production and uncontrolled IL‐6R signaling, on the other hand, contribute to disease development [26]. Tocilizumab and sarilumab, which are anti‐IL‐6R antibodies, have demonstrated success in blocking IL‐6 signaling in RA therapy [27]. YTHDF2 directly recognizing the m6A modification on IL‐6R can decrease the stability of IL‐6R mRNA, thus mitigating sepsis by inhibiting inflammation [28]. However, there is currently no robust evidence to support whether similar mechanisms can lead to therapeutic effects in RA.

This study explored the expression patterns and molecular mechanisms of YTHDF2 and IL‐6R in RA and speculated that YTHDF2 may be a potential therapeutic target for the RA inflammatory response and bone destruction. Our findings provide compelling evidence that elevated YTHDF2 levels inhibit inflammatory responses, proliferation, migration, and invasion of RA FLS. Furthermore, we demonstrate for the first time that intra‐articular injection of YTHDF2‐overexpressing lentivirus attenuates arthritis severity in CIA models. Intriguingly, our results showed that IL‐6R mRNA is modified by m6A and is recognized by YTHDF2. The protective effects of YTHDF2, observed both in vitro and in vivo, can be reversed by overexpression of IL‐6R, suggesting that YTHDF2's ability to bind m6A‐marked IL‐6R mRNA and regulate its expression is functionally significant. Collectively, our data indicate that YTHDF2 may play an essential role in mitigating synovial fibroblast inflammation and bone injury in the pathogenesis of RA by reducing the mRNA stability of IL‐6R, providing a theoretical evidence for exploring novel therapeutic approaches for RA.

## 2 Materials and Methods

### 2.1 Patients

A total of 50 RA patients admitted to People's Hospital of Ningxia Hui Autonomous Region from 2019 to 2022 and 26 age‐matched healthy controls were recruited to collect synovial tissues. RA patients were newly diagnosed, and their clinical diagnosis met the 2010 rheumatoid arthritis classification criteria [29]. No corticosteroids or immunosuppressive drugs were given to them before blood samples were collected. Healthy control subjects had no history of arthritis. Patients with other autoimmune diseases, inflammation, hormone diseases, cancer or mental disorders were excluded. This study was approved and supervised by the ethics committee of People's Hospital of Ningxia Hui Autonomous Region. All subjects signed informed consent and enjoyed the right to know.

### 2.2 Cell Culture and Transfection

Human FLS MH7A cells (ATCC, VA, USA) and HEK293T cells (National Collection of Authenticated Cell Cultures, Shanghai, China) were cultured in DMEM (Gibco, NY, USA) containing 10% fetal bovine serum (Gibco) and 1% penicillin–streptomycin (Gibco) at 37°C and 5% CO_2_ saturated humidity.

### 2.3 Cell Transfection

YTHDF2 overexpression plasmid (OE‐YTHDF2), IL‐6R overexpression plasmid (OE‐IL‐6R), YTHDF2 shRNA (sh‐YTHDF2), IL‐6R shRNA (sh‐IL‐6R) and the corresponding negative controls were synthesized by GenePharma (Shanghai, China).

MH7A cells, planted in 6‐well plates, were grown until they reached 60% confluence for transfection, utilizing Lipofectamine 3000 (Invitrogen, CA, USA). The transfection efficacy was confirmed using RT‐qPCR or Western blot techniques. Post 48 h, cells were treated with 24‐h induction with TNF‐α (10 ng/mL, PeproTech, NJ, USA) to induce inflammation [30, 31].

### 2.4 MTT Assay

Cells were made into a single cell suspension and inoculated in 96‐well plates at 2 × 10^5^/mL, 100 μL per well for 48 h. Then, cells were placed in a serum‐free culture medium for 12 h and incubated with MTT (5 g/L, Beyotime, Shanghai, China) at 10 μL/well for 4 h. Formanzan was added at 100 μL/well and completely dissolved under an optical microscope. The absorbance value was measured at 570 nm using a microplate reader.

### 2.5 EdU Assay

Cells were inoculated into 96‐well plates at 6000 cells/well and continued to be cultured for 24 h. When the cell confluence reached 60%, 100 μL diluted EdU solution was added to each well and continued to be incubated for 2 h. According to the instructions of EdU kit (RiBobio, Guangzhou, China), cells were fixed and stained. Three fields of view were randomly selected and the cells were counted by ImageJ software.

### 2.6 Flow Cytometry

Cells were digested with 0.25% trypsin (without EDTA) and centrifuged twice. According to the instructions of Annexin‐V‐FITC apoptosis detection kit (Beyotime, Shanghai, China), 500 μL loading buffer was mixed with cells, and then 5 μL Annexin V‐FITC and 10 μL PI solution were incubated at room temperature in the dark for 15–20 min, and the apoptosis rate was detected on a flow cytometer (BD Biosciences, NJ, USA).

### 2.7 Scratch Test

Cells were inoculated into 6‐well plates (3 × 10^5^ cells/well). After 24 h, scratches were made on the cell layer using the sterile 200 μL tip. Non‐adherent cells were removed by PBS washing. The gap left after scratch was clearly visible, and then a fresh medium was replaced. Cells were observed at 0 and 48 h, respectively to measure scratch width under a microscope (×40).

### 2.8 Transwell Assay

A Transwell‐chamber culture system (Becton Dickinson, NJ, USA) was employed to detect the migration ability of cells, and Matrigel coating was particularly needed for detecting cellular invasion ability. Cells were digested, washed twice with serum‐free medium, and resuspended. The upper chamber received about 200 μL of cell suspension (1 × 10^5^ cells), and the lower chamber was filled with 500 μL of DMEM containing 10% FBS (20 ng/mL). After 24 h, cells in the lower chamber were fixed with 4% paraformaldehyde solution for 10 min, followed by crystal violet staining. Cells were observed under an optical microscope and counted using ImageJ software.

### 2.9 Experimental Animals

A total of 64 male DBA/1 mice, aged 8 weeks and weighing between 21 and 22 g, were obtained from Shanghai Jiesijie Laboratory Animal Co. Ltd. in Shanghai, China. They were housed individually, with unrestricted access to food and water, under a 12‐h light cycle, a temperature range of 23°C–26°C, and relative humidity controlled at 40%–70%. One week of adaptive feeding was given to the mice. This experiment was reviewed by the Animal Ethics Committee of People's Hospital of Ningxia Hui Autonomous Region.

### 2.10 Preparation of CIA Model

A mouse CIA model was established [32, 33]. Collagen type II (2 mg/mL, Chondrex, USA) was dissolved overnight with 0.1 M acetic acid at 4°C and emulsified with an equal volume of complete adjuvant (Sigma‐Aldrich, MO, USA) containing 2 mg/mL inactivated BCG. DBA/1 mice were injected with 100 μL (2 mg/mL) emulsified collagen (Day 0) via the tail vein. After 21 days (Day 21), 100 μL emulsified collagen was injected again.

### 2.11 Treatments in Mice

The overexpression plasmid targeting YTHDF2/IL‐6R was inserted into the corresponding lentiviral vector (GenePharma). Then the vector and plasmid were co‐transfected into HEK293T cells. The supernatant containing the liberated lentiviral vector was gathered, condensed through ultracentrifugation, and calibrated to a concentration of 2 × 10^8^ TU/mL.

A total of 48 mice were randomly divided into 6 groups (*n* = 8): Non‐treated animals (Naïve), model group (CIA group), Lv‐NC group, Lv‐YTHDF2 group, Lv‐YTHDF2 + Lv‐NC group, and Lv‐YTHDF2 + Lv‐IL‐6R group. From Day 28, mice in the Lv‐NC group, Lv‐YTHDF2 group, Lv‐YTHDF2 + Lv‐NC group, and Lv‐YTHDF2 + Lv‐IL‐6R group were injected with corresponding lentivirus solution (5 × 10^8^TU/mL, once every 4 days) into the ankle joint, and mice in the Naïve group and CIA group were injected with an equal volume of PBS as the control.

After Day 21, the mice were scored for RA every week. A qualitative scoring system was used to evaluate the severity of inflammation [34], 0 = *normal*; 1 = *mild inflammation*, manifested as obvious redness and swelling of the ankle or wrist or obvious redness and swelling of a single finger or toe, regardless of the number affected; 2 = *moderate inflammation*, obvious swelling around the ankle or wrist; 3 = *severe inflammation*, including redness and swelling of the fingers or toes; 4 = *maximum inflammation*, extensive involvement of multiple joints. The aggregate score for arthritis was determined by summing the scores from all four paws, capping at 16 points. The scoring method was blinded and completed by three independent investigators. Mice were allowed to eat and drink water freely and were closely monitored every day. On Day 49, the mice were euthanized by inhaling carbon dioxide.

### 2.12 Serum and Tissue Specimens

Venous blood from the abdominal aorta was taken before the mice were euthanized and centrifuged at 3000 r/min at 4°C for 15 min, and the serum was separated and stored for subsequent ELISA detection. After euthanizing the mice, their ankle joints were fixed in 4% paraformaldehyde, decalcified with 10% EDTA, dehydrated using a series of alcohols, cleared with xylene, embedded in paraffin, and then sliced. Furthermore, a segment of the synovial tissue was preserved in liquid nitrogen for analysis using RT‐qPCR and Western blot techniques.

### 2.13 HE Staining

Paraffin slices were dehydrated by gradient alcohols and cleared with xylene. Then, the slices were stained with hematoxylin solution for 3–5 min, differentiated with 1% hydrochloric acid alcohol for 20 s, and immersed in 1% ammonia for 30 s. Next, the slices were re‐stained with 1% eosin solution for 5 min, followed by conventional dehydration and clearance (75% ethanol for 5 min, 90% ethanol for 5 min, 95% ethanol for 5 min, absolute ethanol for 5 min, and xylene for 10 min × 2 times). The morphological structure of the tissue was observed under an optical microscope (Olympus, Tokyo, Japan). Synovial inflammation and hyperplasia were assessed. 0 = *no inflammation* and synovial cell proliferation; 1 = *mild synovial cell proliferation*, defined as synovial cell thickness of less than 4 layers or a small amount of scattered inflammatory cell infiltration; 2 = *mild synovial cell proliferation* and limited scattered inflammatory cell infiltration; 3 = *moderate synovial cell proliferation*, synovial cell thickness of more than 4 layers, joint space reduction, accompanied by moderate inflammatory cell infiltration, inflammatory cell aggregation of more than 2; 4 = *severe synovial cell proliferation*, manifested as joint space occlusion, inflammatory cells widely infiltrated into the synovial space.

### 2.14 Safranin O/Fast Green Staining

Paraffin slices were dehydrated at 65°C for 4 h and dewaxed with different concentrations of xylene and ethanol. Hematoxylin was added dropwise for 1–3 min, and hydrochloric acid ethanol was added for differentiation for 20 s. The extracellular matrix was stained with 0.02% fast green solution (HXBio, Hangzhou, China) for 3 min and washed with 1% glacial acetic acid for 3 times, and 0.1% safranin O solution (HXBio) was added. After 3 min, the slices were dehydrated with 95% ethanol and absolute ethanol, permeabilized with xylene, sealed with neutral gum, and observed under an optical microscope (Olympus). Cartilage injury was evaluated. 0 signifies a smooth joint surface without erosion; 1 indicates minimal bone erosion, marked by a single, minor, and superficial erosion site; 2 denotes slight bone erosion, with 2–4 points visible in a confined region and marked by dim tones; 3 represents moderate bone erosion with ulcers on the articular surface, where over 5 erosion points penetrate the deep cartilage; and 4 denotes intense bone erosion, with cortical bone fully eroded and subchondral bone uncovered.

### 2.15 TRAP Staining

Paraffin sections were dewaxed, and TRAP staining was performed using a TRAP staining kit (SaiFang Biological, China). Slices were dehydrated, permeabilized, and sealed with neutral gum. Osteoclast staining was observed under an optical microscope (Olympus), and TRAP‐positive cells were counted using ImageJ software.

### 2.16 Immunohistochemistry

Tissue sections were routinely dewaxed and hydrated. The endogenous peroxidase was removed using H_2_O_2_, followed by antigen retrieval via microwave. Following a 30‐min blockage using 10% standard goat serum, TNF‐α (1:500, Abcam, Cambridge, UK), IL‐1β (1:500, Abcam), and IL‐6 (1:600, Abcam) antibodies were incubated for 1 h. Subsequently, the secondary antibody was introduced and incubated for another 30 min. Once DAB color was developed, hematoxylin counterstaining was done, followed by sealing with neutral resin. Five views of the field were randomly selected for 400x magnification photography, and the count of positive cells was determined using ImageJ software.

### 2.17 Elisa

TNF‐α, IL‐1β, and IL‐6 in mouse serum or cell supernatant were determined using ELISA kits (R&D Systems, MN, USA).

### 2.18 Western Blot

The total protein of tissues and cells was extracted using a protein extraction kit (Thermo Scientific, MA, USA) and quantified by a BCA protein concentration detection kit (Thermo Scientific). Electrophoresis was performed according to 40 μg proteins per well. The protein was transferred to PVDF membranes, sealed with 5% skimmed milk powder for 1 h, and combined with YTHDF2 (1: 1000, ab220163, rabbit monoclonal, Abcam), IL‐6R(1:1000, ab300581, rabbit monoclonal, Abcam), MMP3 (1:1000, ab52915, rabbit monoclonal, Abcam), MMP9 (1:1000, ab38898, rabbit polyclonal, Abcam) and GAPDH (1:5000, ab9485, rabbit polyclonal, Abcam) at 4°C. The secondary antibody HRP‐conjugated anti‐rabbit/mouse IgG (Sangon Biotech, Shanghai, China) was incubated at room temperature for 1 h. After color development, protein bands were evaluated.

### 2.19 RT‐qPCR

Total RNA was obtained from both tissues and cells utilizing the Trizol kit (Invitrogen). Total RNA levels were quantified using an ultraviolet spectrophotometer (Beckmen), while its integrity was assessed through agarose electrophoresis. The RNA was subjected to reverse transcription into cDNA utilizing the reverse transcription kit (Fermentas, NY, USA). GeneChem (Shanghai, China) was responsible for generating the primer sequences listed in Table 1. Every gene was measured using the fluorescence quantitative PCR kit (Takara, Dalian, China). Reaction parameters included: a 5‐min pre‐denaturation at 95°C, 45‐s denaturation at 94°C, followed by 35 cycles of annealing at 56°C for 45 s and extension at 72°C for 45 s. Samples were identified using real‐time fluorescence quantitative PCR (ABI 7500, ABI, CA, USA). Each target gene was calculated by the 2^‐∆∆Ct^ method with GAPDH as the internal reference.

**TABLE 1:** Primer sequences.

Genes	Sequences (5′‐3′)
homo‐YTHDF2	Forward: GGCAGCACTGAAGTTGGG
	Reverse: CTATTGGAAGCCACGATGTTA
mmu‐YTHDF2	Forward: GGATGGCAGCACTGAAA
	Reverse: CTGGTTTTGGAGGAGCAA
homo‐IL‐6R	Forward: CCAGCATCACTGTGTCATCCA
	Reverse: AGGACTCCTGGATTCTGTCCA
mmu‐IL‐6R	Forward: TTGGGTTGCTTCTCTGTGT
	Reverse: AAGGTCGGCTTCAGTGG
homo‐GAPDH	Forward: GAAGGTGAAGGTCGGAGTC
	Reverse: GAAGATGGTGATGGGATTTC
mmu‐GAPDH	Forward: GGGTCCCAGCTTAGGTTCAT
	Reverse: CCCAATACGGCCAAATCCGT

Abbreviations: GAPDH, glyceraldehyde 3‐phosphate dehydrogenase; IL‐6R, interleukin‐6 receptor; YTHDF2, YTH domain family 2.

### 2.20 RNA Immunoprecipitation (RIP) Assay

RIP experiments were performed with the Magna RIP kit (Millipore, MA, USA). Cells were lysed using a complete RIP lysis buffer, incubated overnight at 4°C with beads coated with anti‐YTHDF2 (ab220163, Abcam), m6A (ab208577, Abcam), and IgG. RNA was extracted, and the enrichment level of IL‐6R on the probe was identified by RT‐qPCR.

### 2.21 Dual Luciferase Reporter Gene Assay

The potential m6A site of IL‐6R was predicted by the bioinformatics website SRAMP. The IL‐6R wild‐type (WT) plasmid containing the m6A site (IL‐6R‐WT) was synthesized. The IL‐6R mutant (MUT) plasmid (IL‐6R‐MUT) was constructed by mutating the binding site. The procedures were performed using a plasmid extraction kit (Promega, USA). Lipofectamine 3000 (Invitrogen) was used to transfect the IL‐6R‐WT or IL‐6R‐MUT plasmid and the YTHDF2 overexpression plasmid (OE‐YTHDF2) or the corresponding negative control into the cells. After 48 h, the cells were lysed, and the luciferase activity was detected by a luciferase detection kit (BioVision, CA, USA).

### 2.22 Detection of mRNA Stability

Cells were cultured for 24 h and treated with 4 μmol/L actinomycin D (Sigma‐Aldrich). The cells were collected at 0, 2, 4, and 8 h, respectively. RNA was extracted, and IL‐6R mRNA was detected by RT‐qPCR.

### 2.23 Statistical Analysis

The data were analyzed by GraphPad Prism 8.0 (GraphPad Software, CA, USA). The data were expressed in the form of mean ± standard deviation, and the Pearson test was employed for correlation analysis. The comparison between the two groups was done using a *t*‐test. One‐way anova, followed by Tukey's multiple comparisons test, was utilized for comparisons between multiple groups. *p* < 0.05 indicated that the difference was statistically significant.

## 3 Results

### 3.1 YTHDF2 Is Downregulated in RA

**FIGURE 1:**
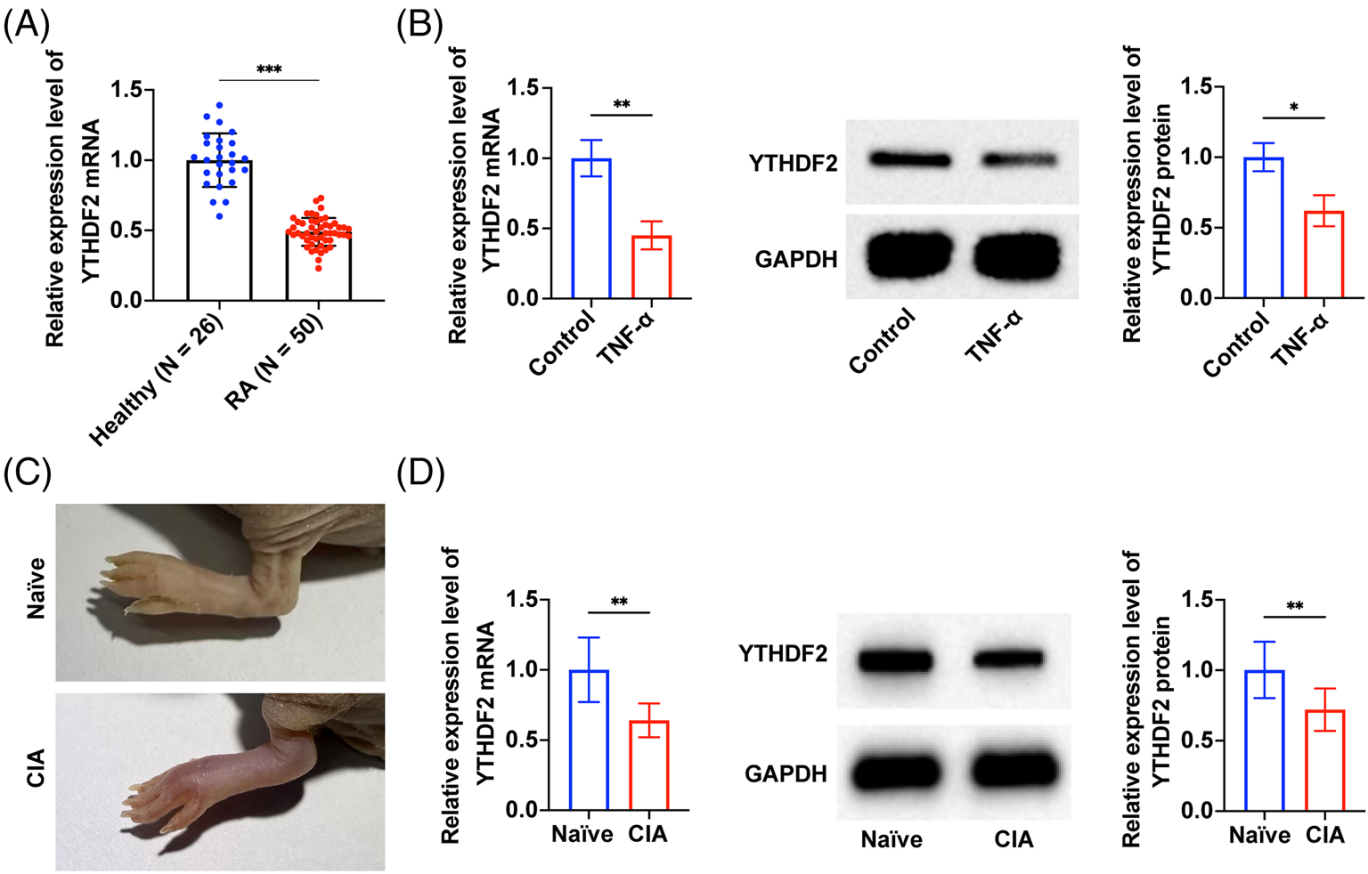
YTHDF2 is downregulated in RA. (A) RT‐qPCR to detect the expression level of YTHDF2 in synovial tissues from 50 RA patients and 26 healthy controls; (B) RT‐qPCR and Western blot to detect the expression level of YTHDF2 in MH7A cells with or without TNF‐α stimulation; (C) Representative image of mice hind limbs exemplify the swelling and ankylosis in CIA mice; (D) RT‐qPCR and Western blot to detect the expression level of YTHDF2 in synovial tissue of CIA mice.

YTHDF2 in synovial tissues of RA patients was significantly down‐regulated (Figure 1A). The same expression trend was also observed in TNF‐α‐induced human MH7A cells (Figure 1B). Furthermore, a CIA model was successfully induced in mice to investigate the potential role of YTHDF2 on RA in vivo (Figure 1C). RT‐qPCR and Western blot analyses revealed a reduction in YTHDF2 expression in CIA mice (Figure 1D).

### 3.2 YTHDF2 Inhibits Inflammation and Proliferation of RA FLS

**FIGURE 2:**
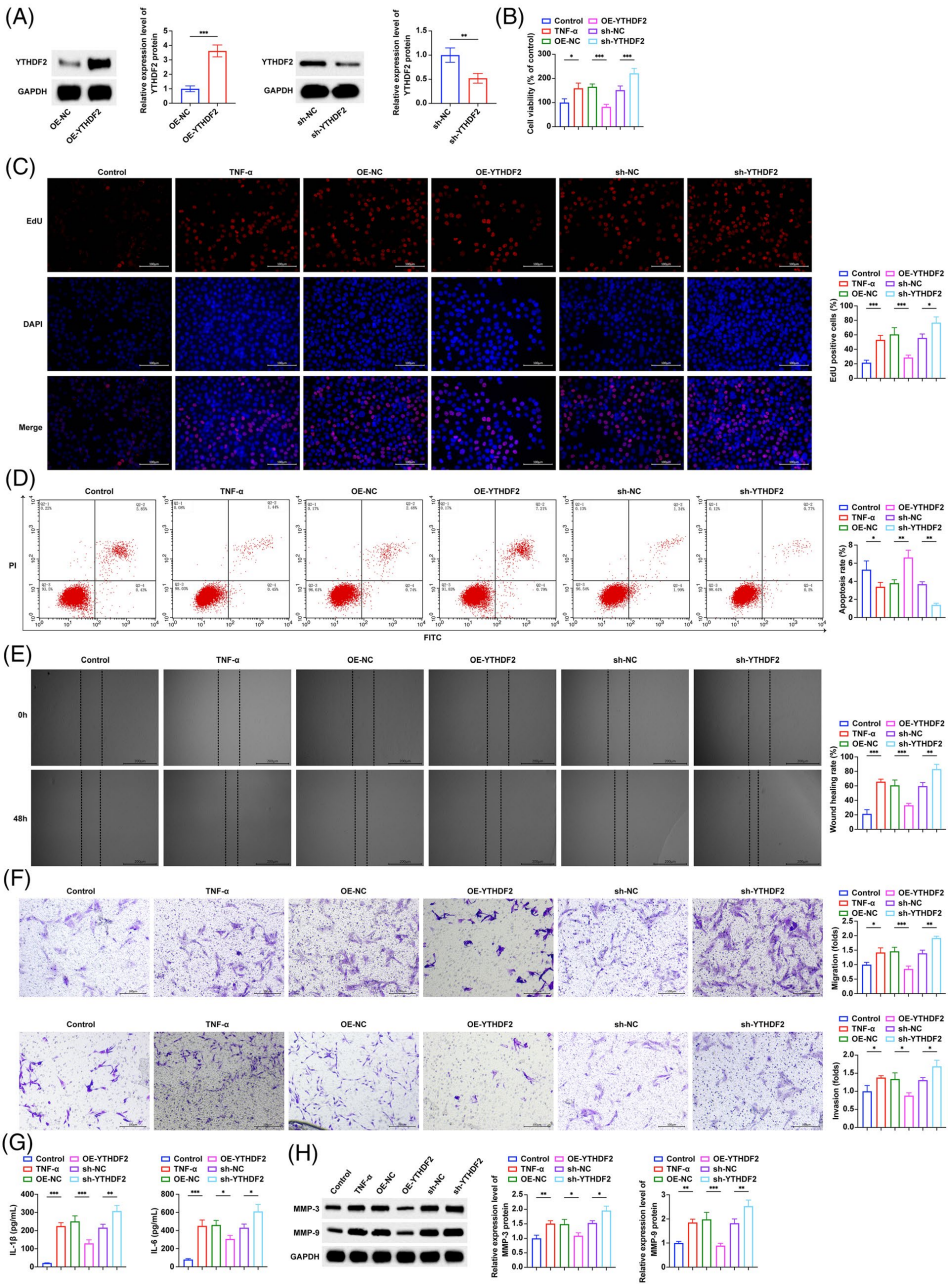
YTHDF2 inhibits inflammation and proliferation of RA FLS. (A) Western blot to verify the efficiency of overexpression and knockdown of YTHDF2; (B–C) MTT and EdU assays to detect the effect of overexpression and knockdown of YTHDF2 on cell viability; (D) Flow cytometry to detect the effect of overexpression and knockdown of YTHDF2 on apoptosis rate; (E–F). Scratch test and Transwell to detect the effect of overexpression and knockdown of YTHDF2 on cell migration and invasion; (G) ELISA to detect the effect of overexpression and knockdown of YTHDF2 on the levels of inflammatory factors IL‐1β and IL‐6 in cell supernatant; (H) Western blot to detect the effect of overexpression and knockdown of YTHDF2 on the expression of MMP3 and MMP9 proteins in cells. *p < 0.05, **p < 0.01, ***p < 0.001. At least three repeated experiments were conducted.

The transfection efficiency of the YTHDF2 overexpression plasmid, YTHDF2 shRNA, and corresponding negative controls was verified by Western blot (Figure 2A). MTT and EdU experiments (Figure 2B,C) showed that the increased viability and proliferation of MH7A cells induced by TNF‐α were inhibited after overexpression of YTHDF2 and further increased after knockdown of YTHDF2. Flow cytometry showed that the apoptosis rate of MH7A cells decreased after TNF‐α induction. Overexpressing YTHDF2 promoted apoptosis, while knockdown of YTHDF2 inhibited apoptosis (Figure 2D). Scratch test and Transwell manifested that (Figure 2E,F) elevating YTHDF2 reversed TNF‐α‐induced cell migration and invasion, while knocking down YTHDF2 showed the opposite effect. The detection of the concentration of inflammatory factors in the cell supernatant (Figure 2G) found that IL‐1β and IL‐6 levels induced by TNF‐α were increased. Overexpressing YTHDF2 down‐regulated inflammatory factors, while knocking down YTHDF2 further up‐regulated the levels of IL‐1β and IL‐6. In addition, Western blot (Figure 2H) found that TNF‐α induction significantly enhanced MMP3 and MMP9 protein expression, but upregulating YTHDF2 reversed this promotion, while knockdown of YTHDF2 further up‐regulated MMP3 and MMP9.

### 3.3 YTHDF2 Alleviates Synovial Inflammation and Bone Damage in CIA Mice

**FIGURE 3:**
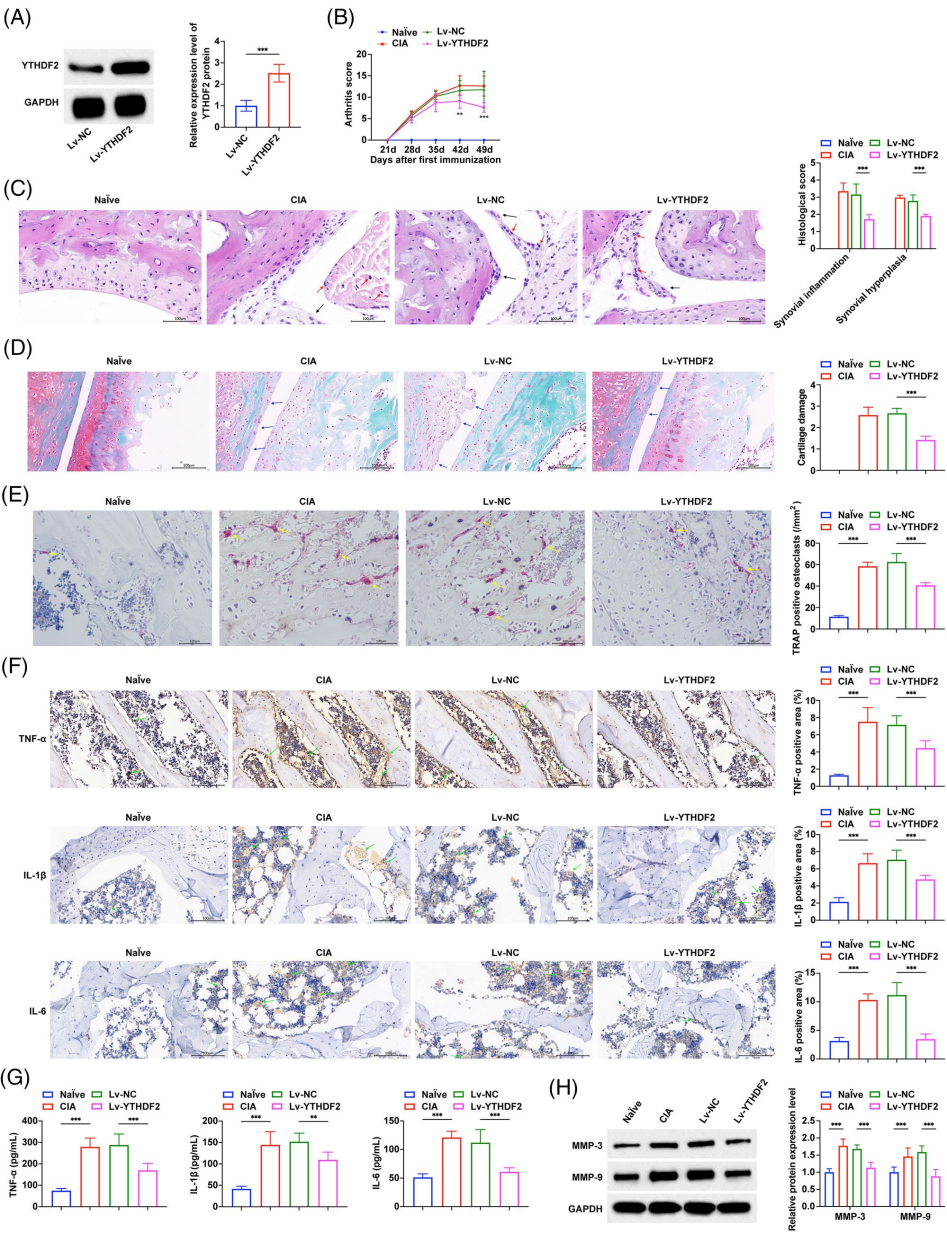
YTHDF2 alleviates synovial inflammation and bone damage in CIA mice. (A) Western blot to confirm the overexpression of YTHDF2 by lentiviral vector; (B) Clinical score of arthritis in mice; (C) HE staining of ankle joint tissues of mice and the quantitative analysis of synovial inflammation and hyperplasia; (D) Quantitative analysis of representative pictures of safranin O/fast green staining and cartilage injury in ankle joint tissues of mice; (E) Quantitative analysis of TRAP staining in ankle joints of mice; (F) Immunohistochemistry to detect the effects of overexpression of YTHDF2 on the expression of TNF‐α, IL‐1β, and IL‐6 in ankle joint tissue of CIA mice; (G) ELISA to detect the effects of overexpression of YTHDF2 on serum TNF‐α, IL‐1β, and IL‐6 levels in CIA mice; H. Western blot to detect the effect of overexpression of YTHDF2 on the expression of MMP3 and MMP9 proteins in synovial tissue of CIA mice. The black arrow indicates synovial hyperplasia, the red arrow indicates inflammatory cells, the blue arrow indicates cartilage erosion, the yellow arrow indicates TRAP positive cells, and the green arrow indicates immunohistochemistry positive area. *n* = 8. *p < 0.05, **p < 0.01, ***p < 0.001. At least three repeated experiments were conducted.

We further studied the effect of YTHDF2 on RA in vivo by intra‐articular injection of YTHDF2 overexpressing lentivirus to intervene in CIA mice. Western blot results showed that lentivirus intervention successfully changed YTHDF2 expression in CIA mice (Figure 3A). Compared with CIA mice, we observed a significant decrease in arthritis severity scores after YTHDF2 overexpression (Figure 3B). The severity of arthritis in CIA mice was further evaluated by HE staining, safranin O/fast green staining, and TRAP staining. In CIA mice, the ankle joints demonstrated synovial hyperplasia and inflammation, extensive joint damage with cartilage erosion, and a significant rise in osteoclast numbers. However, after up‐regulation of YTHDF2, synovial hyperplasia and inflammation, as well as cartilage injury in mice, were significantly improved, and the number of osteoclasts was reduced (Figure 3C–E). TNF‐α, IL‐1β and IL‐6 in the ankle joint of mice were detected by immunohistochemistry. It was found that TNF‐α, IL‐1β, and IL‐6 in CIA mice were significantly down‐regulated after up‐regulating YTHDF2 (Figure 3F). ELISA found that serum TNF‐α, IL‐1β and IL‐6 in CIA mice were significantly higher (Figure 3G), which were reversed by overexpression of YTHDF2. Western blot detection of MMP3 and MMP9 in mouse synovial tissue (Figure 3H) showed that MMP3 and MMP9, which were originally up‐regulated in CIA mice, were significantly inhibited after up‐regulating YTHDF2.

### 3.4 YTHDF2 Reduces IL‐6R mRNA Stability in an m6A‐Dependent Manner

**FIGURE 4:**
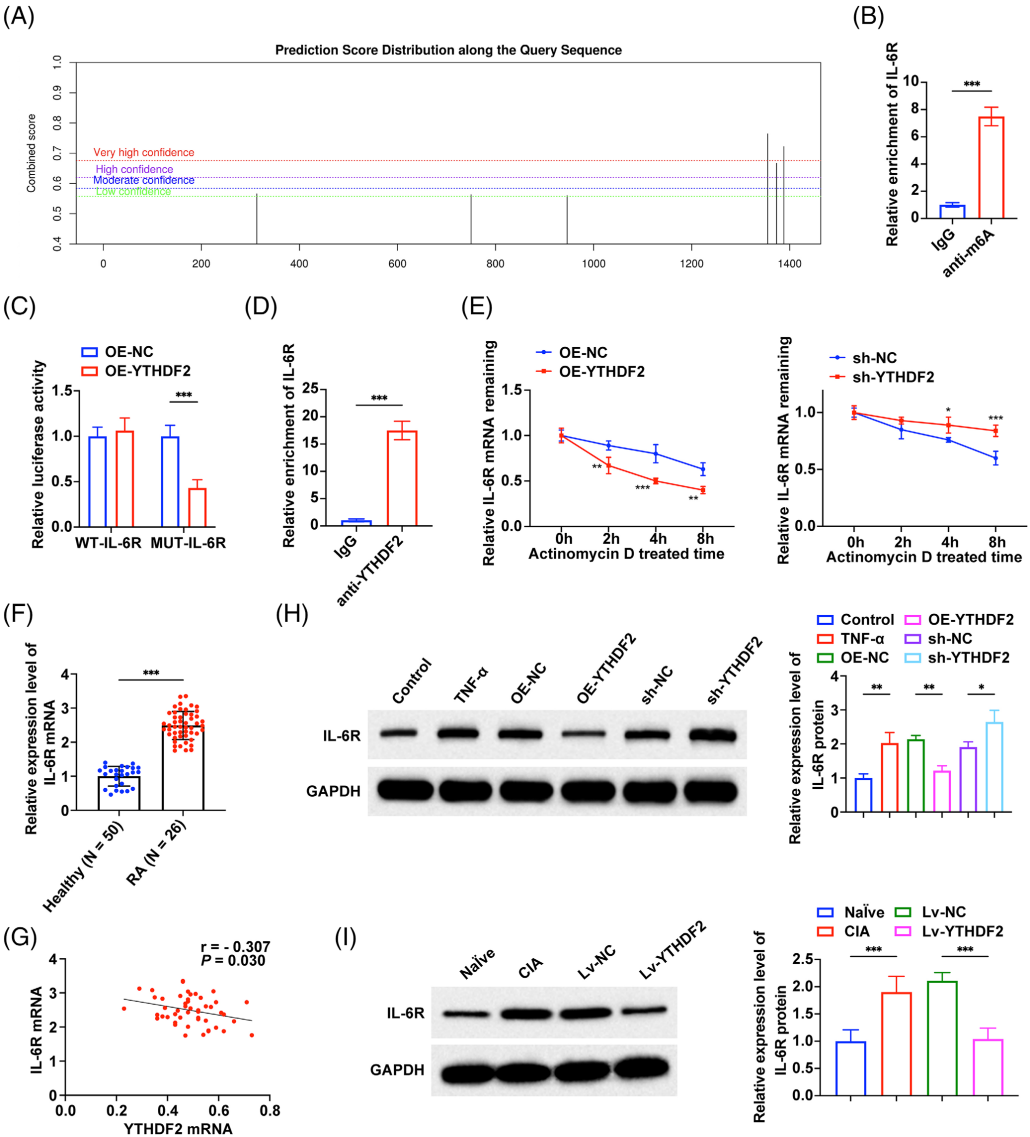
YTHDF2 reduces IL‐6R mRNA stability in a m6A‐dependent manner. (A) SRAMP to predict potential m6A sites of IL‐6R; (B) m6A‐RIP to detect the m6A enrichment of IL‐6R mRNA in MH7A cell line; (C) Dual luciferase reporter gene assay to verify the binding relationship between IL‐6R and YTHDF2 in MH7A cell line; (D) RIP assay to verify the binding relationship between IL‐6R and YTHDF2 in MH7A cell line; (E) MRNA stability assay to detect the effect of overexpression and knockdown of YTHDF2 on the stability of IL‐6R mRNA; (F) RT‐qPCR to detect the expression level of IL‐6R in synovial tissues from 50 RA patients and 26 healthy controls; (G) Pearson test to analyze the correlation between the expression levels of YTHDF2 and IL‐6R in synovial tissue from 50 RA patients; (H) Western blot to detect the protein expression of IL‐6R in MH7A cells; (I) Western blot to detect the protein expression of IL‐6R in synovial tissue of CIA mice, *n* = 8. *p < 0.05, **p < 0.01, ***p < 0.001. At least three repeated experiments were conducted.

We further studied the downstream mechanism of YTHDF2. Starbase (http://starbase.sysu.edu.cn/) predicted that there was a binding site between YTHDF2 and IL‐6R. SRAMP (https://www.cuilab.cn/sramp) was used to predict the modification of IL‐6R mRNA by m6A through the whole transcription model and RNA secondary structure analysis, and the potential m6A modification site of IL‐6R mRNA was obtained (Figure 4A). The m6A‐RIP test results confirmed IL‐6R m6A modification in MH7A cells (Figure 4B). The dual luciferase reporter gene assay showed that overexpressing YTHDF2 significantly reduced the luciferase activity of WT‐IL‐6R but had no significant effect on MUT‐IL‐6R (Figure 4C). The interaction between YTHDF2 and IL‐6R mRNA was further verified by the RIP assay (Figure 4D). We analyzed the mRNA stability of IL‐6R in transfected cells to further study the relationship between YTHDF2 and IL‐6R mRNA degradation (Figure 4E). The results determined that overexpressing YTHDF2 significantly promoted the degradation of IL‐6R mRNA and reduced the mRNA stability of IL‐6R. Down‐regulation of YTHDF2 showed the opposite effect.

IL‐6R was up‐regulated in RA patients, and the Pearson test showed that IL‐6R in RA patients was negatively correlated with YTHDF2 (Figure 4F,G). Similarly, IL‐6R was also significantly up‐regulated in TNF‐α‐induced MH7A cells and CIA mice, and the expression level was negatively regulated by YTHDF2 (Figure 4H,I).

### 3.5 IL‐6R Mediates the Inhibitory Effect of YTHDF2 on Inflammation and Proliferation of RA FLS

**FIGURE 5:**
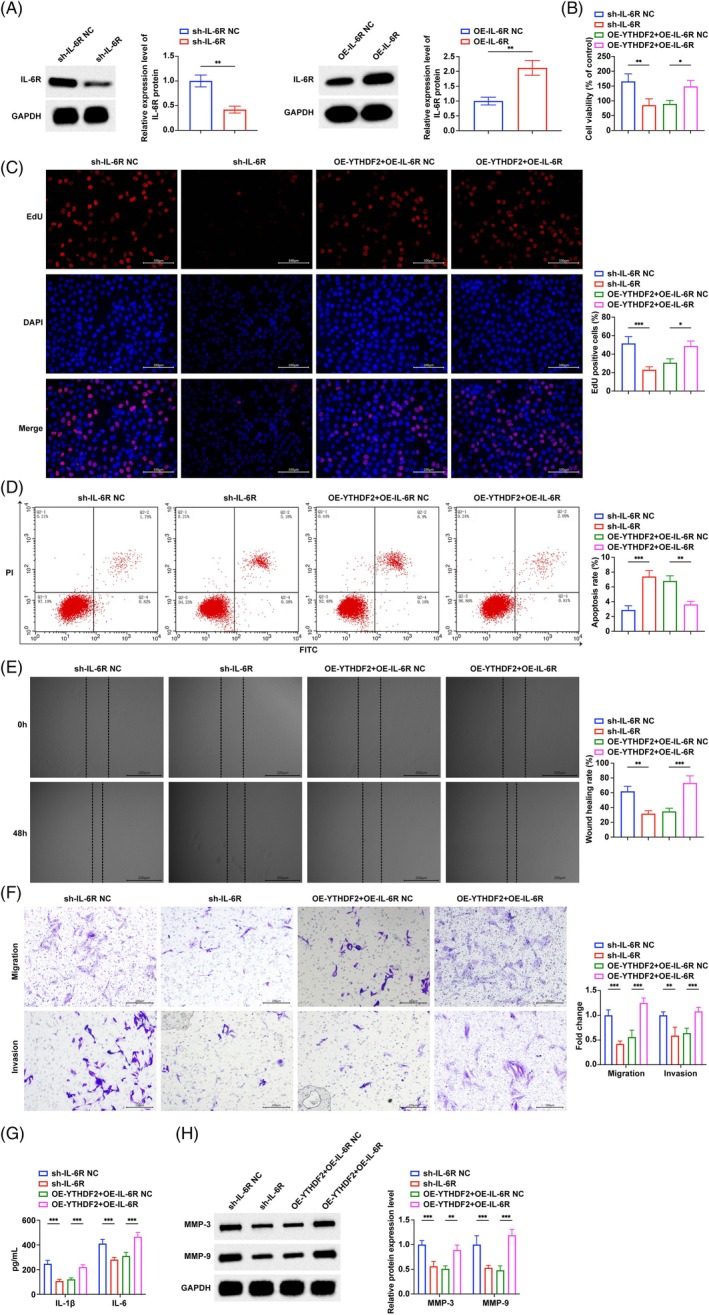
IL‐6R mediates the inhibitory effect of YTHDF2 on the inflammation and proliferation of RA synovial fibroblasts. (A) Western blot to verify the efficiency of overexpression and knockdown of IL‐6R; (B) MTT assay to detect the effect of IL‐6R on cell viability; (C) EdU assay to detect the effect of IL‐6R on cell proliferation; (D) Flow cytometry to detect the effect of IL‐6R on apoptosis rate; (E–F) Scratch test and Transwell to detect the effect of IL‐6R on cell migration and invasion; (G) ELISA to detect the effect of IL‐6R on the levels of inflammatory factors IL‐1β and IL‐6 in cell supernatant; (H) Western blot to detect the effect of IL‐6R on the expression of MMP3 and MMP9 proteins in cells. *p < 0.05, **p < 0.01, ***p < 0.001. At least three repeated experiments were conducted.

Next, we transfected IL‐6R shRNA and the corresponding negative control into TNF‐α‐induced MH7A cells to study the effect of IL‐6R on inflammation and the biological function of RA FLS. The transfection efficiency was verified by RT‐qPCR (Figure 5A). The increased viability and proliferation of MH7A cells induced by TNF‐α were inhibited after the knockdown of IL‐6R (Figure 5B,C). Moreover, knocking down IL‐6R promoted the apoptosis of MH7A cells (Figure 5D). MH7A cells with low expression of IL‐6R showed weaker migration and invasion ability (Figure 5E,F). IL‐1β and IL‐6 induced by TNF‐α were increased, and reducing IL‐6R down‐regulated the inflammatory factors (Figure 5G). Suppressing IL‐6R inhibited MMP3 and MMP9 in MH7A cells induced by TNF‐α (Figure 5H). The rescue experiment further verified the functional interaction between IL‐6R and YTHDF2. In detail, overexpressing IL‐6R effectively impaired the effect of the overexpression of YTHDF2 on the proliferation, migration, invasion, and apoptosis of MH7A cells induced by TNF‐α (Figure 5B–F). Overexpression of IL‐6R also counteracted the effect of the down‐regulation of YTHDF2 on the levels of inflammatory factors and the expression of MMP3 and MMP9 (Figure 5G,H).

### 3.6 IL‐6R Reverses the Improvement of Synovial Inflammation and Bone Damage in CIA Mice by YTHDF2

**FIGURE 6:**
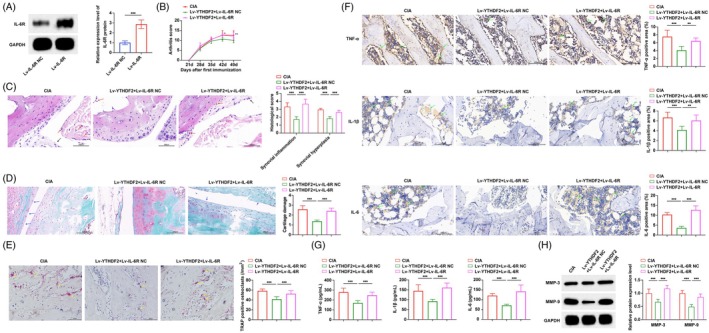
IL‐6R reverses the improvement of synovial inflammation and bone damage in CIA mice by YTHDF2. (A) Western blot to confirm the overexpression of IL‐6R by lentiviral vector; (B) Clinical score of arthritis in mice; (C) HE staining of ankle joint tissues of mice and quantitative analysis of synovial inflammation and hyperplasia; (D) Quantitative analysis of safranin O/fast green staining and cartilage injury in ankle joint tissues of mice; (E) Quantitative analysis of TRAP staining and osteoclasts in ankle joints of mice; (F) Immunohistochemistry to detect the effect of overexpression of IL‐6R on the expression of TNF‐α, IL‐1β, and IL‐6 in ankle joint tissue of CIA mice; (G) ELISA to detect the effects of overexpression of IL‐6R on serum TNF‐α, IL‐1β, and IL‐6 levels in CIA mice; (H) Western blot to detect the effect of overexpression of IL‐6R on the expression of MMP3 and MMP9 proteins in synovial tissue of CIA mice. The black arrow indicates synovial hyperplasia, the red arrow indicates inflammatory cells, the blue arrow indicates cartilage erosion, the yellow arrow indicates TRAP positive cells, and the green arrow indicates immunohistochemistry positive area. *n* = 8. *p < 0.05, **p < 0.01, ***p < 0.001. At least three repeated experiments were conducted.

We also carried out rescue experiments in vivo. Western blot results showed that lentivirus intervention successfully changed IL‐6R levels in CIA mice (Figure 6A). The up‐regulation of IL‐6R weakened the effect of YTHDF2 on the decrease of arthritis severity score in CIA mice (Figure 6B). High expression of IL‐6R counteracted synovial hyperplasia, inflammation, and bone destruction that had been improved by YTHDF2 (Figure 6C–E). TNF‐α, IL‐1β, and IL‐6 in mice were also significantly up‐regulated after the up‐regulation of IL‐6R (Figure 6F). The changes in serum TNF‐α, IL‐1β and IL‐6 levels in mice after the up‐regulation of IL‐6R showed the same trend as the changes in tissue levels (Figure 6G). MMP3 and MMP9, which were originally down‐regulated by YTHDF2, increased after the up‐regulation of IL‐6R (Figure 6H).

## 4 Discussion

Current studies have shown that the pathological process of RA is mainly divided into three stages. The initiation stage is characterized by the activation of immune cells and the production of autoantibodies. The inflammatory response stage is also called the systemic immune response stage. Autoantibodies form immune complexes, and activated lymphocytes and macrophages secrete more inflammatory cytokines, causing a systemic inflammatory response. The main pathological process is that the mesenchymal cells in the affected joints proliferate and differentiate into RA FLS under the stimulation of inflammatory cytokines. RA FLS can promote the formation of osteoclasts through RNAKL, eventually leading to bone and cartilage destruction [35, 36]. Synovitis in the inflammatory response stage is the most basic pathological feature in the course of RA. The inflammatory microenvironment and pro‐inflammatory cytokines induce FLS proliferation in the synovial tissues of RA patients [5]. Proliferated FLS have additional pro‐inflammatory effects, which promote lymphocyte infiltration from plasma to synovium by releasing various cytokines and chemokines, leading to synovitis. RA FLS can also stimulate the formation of osteoclasts by releasing various cytokines, leading to bone and cartilage destruction [37, 38].

This study found that YTHDF2 was down‐regulated in synovial tissues of RA patients, and the same expression trend was also observed in the TNF‐α‐induced human FLS MH7A cell line. This is consistent with the expression trend of YTHDF2 in RA found in the past [20, 21, 22]. To further determine the effect of YTHDF2 on TNF‐α‐induced MH7A cell phenotype, we performed functional experiments after the overexpression or knockdown of YTHDF2 in cells. The results showed that overexpressing YTHDF2 inhibited cell proliferation, migration, invasion, and inflammatory response, and promoted apoptosis. Overexpressing YTHDF2 also inhibited MMP3 and MMP9 protein expression. MMPs are closely related to the invasiveness of RA FLS and are also an important cause of cartilage erosion. Knockdown of YTHDF2 had the opposite effect on cell phenotype compared with the overexpression of YTHDF2. In CIA mice, we verified that overexpressing YTHDF2 effectively improved synovial inflammation and bone damage in CIA mice and delayed disease progression.

After that, we further studied the mechanism of YTHDF2 affecting RA. In RA FLS, the IL‐6R and IL‐6 pathway are affected by a variety of epigenetic changes, emphasizing their importance in the pathogenesis of RA [37, 39, 40]. IL‐6R inhibitors approved for RA, similar to TNF blockers, can influence the function of FLS by inhibiting RANKL production or chemokine expression [5]. Unsurprisingly, our study showed that IL‐6R was overexpressed in synovial tissues of RA patients and TNF‐α‐induced MH7A cell lines. The IL‐6R level in RA patients was negatively correlated with the YTHDF2 level. We verified the binding relationship between YTHDF2 and IL‐6R. Mechanistically, YTHDF2 promoted the degradation of IL‐6R mRNA in MH7A cells in an m6A‐dependent manner and regulated IL‐6R expression. Functionally, overexpression of IL‐6R in vitro eliminated the regulatory effect of up‐regulated YTHDF2 on the phenotype of TNF‐α‐induced MH7A cells, and overexpressing IL‐6R in vivo also reversed the improvement of up‐regulated YTHDF2 on the inflammatory response and bone injury in CIA mice. These results confirm that YTHDF2 plays a protective role in RA synovial fibroblast inflammation and bone injury by inhibiting IL‐6R.

## 5 Conclusion

In general, YTHDF2 is down‐regulated in RA and has anti‐inflammatory and protective effects on RA. Mechanistically, YTHDF2 inhibits the excessive proliferation, invasion, and inflammatory factor levels in RA FLS by reducing the mRNA stability of IL‐6R, thereby improving the RA inflammatory response and bone injury. It is suggested that YTHDF2 is a potentially promising target for the treatment of RA.

## Ethics Statement

The present study was approved by the Ethics Committee of People's Hospital of Ningxia Hui Autonomous Region (No. 201811NX‐06) and written informed consent was provided by all patients prior to the study start. All procedures were performed in accordance with the ethical standards of the Institutional Review Board and The Declaration of Helsinki, and its later amendments or comparable ethical standards. All animal experiments were complied with the ARRIVE guidelines and performed in accordance with the National Institutes of Health Guide for the Care and Use of Laboratory Animals. The experiments were approved by the Institutional Animal Care and Use Committee of People's Hospital of Ningxia Hui Autonomous Region (No. 201905NX‐12).

## Conflicts of Interest

The authors declare no conflicts of interest.

## Data Availability

The data that support the findings of this study are available from the corresponding author upon reasonable request.

## References

[1] E. M. Gravallese and G. S. Firestein , “Rheumatoid Arthritis—Common Origins, Divergent Mechanisms,” New England Journal of Medicine 388, no. 6 (2023): 529–542. 36780677 10.1056/NEJMra2103726

[2] M. H. Smith and J. R. Berman , “What Is Rheumatoid Arthritis?,” JAMA 327, no. 12 (2022): 1194. 35315883 10.1001/jama.2022.0786

[3] F. Rivellese and C. Pitzalis , “Cellular and Molecular Diversity in Rheumatoid Arthritis,” Seminars in Immunology 58 (2021): 101519. 35033462 10.1016/j.smim.2021.101519

[4] G. Nygaard and G. S. Firestein , “Restoring Synovial Homeostasis in Rheumatoid Arthritis by Targeting Fibroblast‐Like Synoviocytes,” Nature Reviews Rheumatology 16, no. 6 (2020): 316–333. 32393826 10.1038/s41584-020-0413-5 PMC7987137

[5] V. Tsaltskan and G. S. Firestein , “Targeting Fibroblast‐Like Synoviocytes in Rheumatoid Arthritis,” Current Opinion in Pharmacology 67 (2022): 102304. 36228471 10.1016/j.coph.2022.102304 PMC9942784

[6] B. Grillet , R. V. S. Pereira , J. Van Damme , A. Abu El‐Asrar , P. Proost , and G. Opdenakker , “Matrix Metalloproteinases in Arthritis: Towards Precision Medicine,” Nature Reviews Rheumatology 19, no. 6 (2023): 363–377. 37161083 10.1038/s41584-023-00966-w

[7] Z. Wu , D. Ma , H. Yang , et al., “Fibroblast‐Like Synoviocytes in Rheumatoid Arthritis: Surface Markers and Phenotypes,” International Immunopharmacology 93 (2021): 107392. 33529910 10.1016/j.intimp.2021.107392

[8] E. Neumann , C. Heck , and U. Müller‐Ladner , “Recent Developments in the Synovial Fibroblast Pathobiology Field in Rheumatoid Arthritis,” Current Opinion in Rheumatology 36, no. 1 (2024): 69–75. 37720975 10.1097/BOR.0000000000000978

[9] R. Ai , T. Laragione , D. Hammaker , et al., “Comprehensive Epigenetic Landscape of Rheumatoid Arthritis Fibroblast‐Like Synoviocytes,” Nature Communications 9, no. 1 (2018): 1921. 10.1038/s41467-018-04310-9 PMC5953939 29765031

[10] S. Guo , L. Xu , C. Chang , R. Zhang , Y. Jin , and D. He , “Epigenetic Regulation Mediated by Methylation in the Pathogenesis and Precision Medicine of Rheumatoid Arthritis,” Frontiers in Genetics 11 (2020): 811. 32849810 10.3389/fgene.2020.00811 PMC7417338

[11] A. Torres , B. Pedersen , I. Cobo , et al., “Epigenetic Regulation of Nutrient Transporters in Rheumatoid Arthritis Fibroblast‐Like Synoviocytes,” Arthritis & Rhematology 74, no. 7 (2022): 1159–1171. 10.1002/art.42077 PMC9246826 35128827

[12] W. Huang , T. Q. Chen , K. Fang , Z. C. Zeng , H. Ye , and Y. Q. Chen , “N6‐Methyladenosine Methyltransferases: Functions, Regulation, and Clinical Potential,” Journal of Hematology & Oncology 14, no. 1 (2021): 117. 34315512 10.1186/s13045-021-01129-8 PMC8313886

[13] X. Jiang , B. Liu , Z. Nie , et al., “The Role of m6A Modification in the Biological Functions and Diseases,” Signal Transduction and Targeted Therapy 6, no. 1 (2021): 74. 33611339 10.1038/s41392-020-00450-x PMC7897327

[14] S. Zaccara , R. J. Ries , and S. R. Jaffrey , “Reading, Writing and Erasing mRNA Methylation,” Nature Reviews Molecular Cell Biology 20, no. 10 (2019): 608–624. 31520073 10.1038/s41580-019-0168-5

[15] L. Chen , Y. Gao , S. Xu , et al., “N6‐Methyladenosine Reader YTHDF Family in Biological Processes: Structures, Roles, and Mechanisms,” Frontiers in Immunology 14 (2023): 1162607. 36999016 10.3389/fimmu.2023.1162607 PMC10043241

[16] J. Li , H. Xie , Y. Ying , et al., “YTHDF2 Mediates the mRNA Degradation of the Tumor Suppressors to Induce AKT Phosphorylation in N6‐Methyladenosine‐Dependent Way in Prostate Cancer,” Molecular Cancer 19, no. 1 (2020): 152. 33121495 10.1186/s12943-020-01267-6 PMC7599101

[17] R. Liu , Y. Jia , G. Kong , and A. He , “Novel Insights Into Roles of N6‐Methyladenosine Reader YTHDF2 in Cancer Progression,” Journal of Cancer Research and Clinical Oncology 148, no. 9 (2022): 2215–2230. 35763107 10.1007/s00432-022-04134-7 PMC11800943

[18] Y. Xu , W. Liu , and L. Ren , “Emerging Roles and Mechanism of m6A Methylation in Rheumatoid Arthritis,” Biomedicine & Pharmacotherapy 170 (2024): 116066. 38157641 10.1016/j.biopha.2023.116066

[19] X. Zhou , Y. Wu , Y. Song , B. Wang , Y. Cai , and C. Miao , “Mechanistic and Therapeutic Insights Into the Function of N6‐Methyladenosine in Arthritic Diseases,” Inflammation Research 74, no. 1 (2025): 7. 39762508 10.1007/s00011-024-01969-3

[20] F. Yao , C. Xu , Y. Gao , et al., “Expression and Clinical Significance of the m6A Reader YTHDF2 in Peripheral Blood Mononuclear Cells From Rheumatoid Arthritis Patients,” Journal of Immunotoxicology 19, no. 1 (2022): 53–60. 35776431 10.1080/1547691X.2022.2067916

[21] J. Xiao , X. Cai , R. Wang , W. Zhou , and Z. Ye , “ALKBH5‐YTHDF2 m6A Modification Axis Inhibits Rheumatoid Arthritis Progression by Suppressing NLRP3,” Biochemical and Biophysical Research Communications 668 (2023): 70–76. 37244037 10.1016/j.bbrc.2023.05.087

[22] G. Li , Y. Fang , N. Xu , Y. Ding , and D. Liu , “Fibroblast‐Like Synoviocytes‐Derived Exosomal circFTO Deteriorates Rheumatoid Arthritis by Enhancing N6‐Methyladenosine Modification of SOX9 in Chondrocytes,” Arthritis Research & Therapy 26, no. 1 (2024): 56. 38388473 10.1186/s13075-024-03290-0 PMC10882813

[23] Z. Chen , A. Bozec , A. Ramming , and G. Schett , “Anti‐Inflammatory and Immune‐Regulatory Cytokines in Rheumatoid Arthritis,” Nature Reviews Rheumatology 15, no. 1 (2019): 9–17. 30341437 10.1038/s41584-018-0109-2

[24] P. Uciechowski and W. C. M. Dempke , “Interleukin‐6: A Masterplayer in the Cytokine Network,” Oncology 98, no. 3 (2020): 131–137. 31958792 10.1159/000505099

[25] S. Kaur , Y. Bansal , R. Kumar , and G. Bansal , “A Panoramic Review of IL‐6: Structure, Pathophysiological Roles and Inhibitors,” Bioorganic & Medicinal Chemistry 28, no. 5 (2020): 115327. 31992476 10.1016/j.bmc.2020.115327

[26] T. Kishimoto and S. Kang , “IL‐6 Revisited: From Rheumatoid Arthritis to CAR T Cell Therapy and COVID‐19,” Annual Review of Immunology 40 (2022): 323–348. 10.1146/annurev-immunol-101220-023458 35113729

[27] Y. J. Lin , M. Anzaghe , and S. Schülke , “Update on the Pathomechanism, Diagnosis, and Treatment Options for Rheumatoid Arthritis,” Cells 9, no. 4 (2020): 880. 32260219 10.3390/cells9040880 PMC7226834

[28] Z. Zeng , Y. Lan , L. Zhang , et al., “The m6A Reader YTHDF2 Alleviates the Inflammatory Response by Inhibiting IL‐6R/JAK2/STAT1 Pathway‐Mediated High‐Mobility Group Box‐1 Release,” Burns Trauma 11 (2023): tkad023. 38026444 10.1093/burnst/tkad023 PMC10650363

[29] D. Aletaha , T. Neogi , A. J. Silman , et al., “2010 Rheumatoid Arthritis Classification Criteria: An American College of Rheumatology/European League Against Rheumatism Collaborative Initiative,” Annals of the Rheumatic Diseases 69, no. 9 (2010): 1580–1588. 20699241 10.1136/ard.2010.138461

[30] L. Kong , L. Wang , Q. Zhao , G. Di , and H. Wu , “Rhodojaponin II Inhibits TNF‐α‐Induced Inflammatory Cytokine Secretion in MH7A Human Rheumatoid Arthritis Fibroblast‐Like Synoviocytes,” Journal of Biochemical and Molecular Toxicology 34, no. 10 (2020): e22551. 32613688 10.1002/jbt.22551

[31] P. Hui , S. Zhou , C. Cao , W. Zhao , L. Zeng , and X. Rong , “The Elucidation of the Anti‐Inflammatory Mechanism of EMO in Rheumatoid Arthritis Through an Integrative Approach Combining Bioinformatics and Experimental Verification,” Frontiers in Pharmacology 14 (2023): 1195567. 37324499 10.3389/fphar.2023.1195567 PMC10267444

[32] Q. Zhang , D. Dehaini , Y. Zhang , et al., “Neutrophil Membrane‐Coated Nanoparticles Inhibit Synovial Inflammation and Alleviate Joint Damage in Inflammatory Arthritis,” Nature Nanotechnology 13, no. 12 (2018): 1182–1190. 10.1038/s41565-018-0254-4 30177807

[33] Y. Xu , Z. Zai , Z. Lu , et al., “Circular RNA CircCDKN2B‐AS_006 Promotes the Tumor‐Like Growth and Metastasis of Rheumatoid Arthritis Synovial Fibroblasts by Targeting the miR‐1258/RUNX1 Axis,” International Journal of Molecular Sciences 24, no. 6 (2023): 5880. 36982956 10.3390/ijms24065880 PMC10051600

[34] Y. Lin , Z. Cheng , Y. Zhong , et al., “Extracorporeal Photopheresis Reduces Inflammation and Joint Damage in a Rheumatoid Arthritis Murine Model,” Journal of Translational Medicine 22, no. 1 (2024): 305. 38528553 10.1186/s12967-024-05105-x PMC10962138

[35] J. Zhao , S. Guo , S. J. Schrodi , and D. He , “Molecular and Cellular Heterogeneity in Rheumatoid Arthritis: Mechanisms and Clinical Implications,” Frontiers in Immunology 12 (2021): 790122. 34899757 10.3389/fimmu.2021.790122 PMC8660630

[36] Q. Ding , W. Hu , R. Wang , et al., “Signaling Pathways in Rheumatoid Arthritis: Implications for Targeted Therapy,” Signal Transduction and Targeted Therapy 8, no. 1 (2023): 68. 36797236 10.1038/s41392-023-01331-9 PMC9935929

[37] M. Masoumi , H. Bashiri , H. Khorramdelazad , et al., “Destructive Roles of Fibroblast‐Like Synoviocytes in Chronic Inflammation and Joint Damage in Rheumatoid Arthritis,” Inflammation 44, no. 2 (2021): 466–479. 33113036 10.1007/s10753-020-01371-1

[38] M. Kugler , M. Dellinger , F. Kartnig , et al., “Cytokine‐Directed Cellular Cross‐Talk Imprints Synovial Pathotypes in Rheumatoid Arthritis,” Annals of the Rheumatic Diseases 82, no. 9 (2023): 1142–1152. 37344156 10.1136/ard-2022-223396

[39] A. K. Singh , M. Haque , B. Madarampalli , et al., “Ets‐2 Propagates IL‐6 Trans‐Signaling Mediated Osteoclast‐Like Changes in Human Rheumatoid Arthritis Synovial Fibroblast,” Frontiers in Immunology 12 (2021): 746503. 34795667 10.3389/fimmu.2021.746503 PMC8593237

[40] R. A. Symons , F. Colella , F. L. Collins , et al., “Targeting the IL‐6‐Yap‐Snail Signalling Axis in Synovial Fibroblasts Ameliorates Inflammatory Arthritis,” Annals of the Rheumatic Diseases 81, no. 2 (2022): 214–224. 34844926 10.1136/annrheumdis-2021-220875 PMC8762018

